# Exogenous Calcium Alleviates Photoinhibition of PSII by Improving the Xanthophyll Cycle in Peanut (*Arachis Hypogaea*) Leaves during Heat Stress under High Irradiance

**DOI:** 10.1371/journal.pone.0071214

**Published:** 2013-08-07

**Authors:** Sha Yang, Fang Wang, Feng Guo, Jing-Jing Meng, Xin-Guo Li, Shu-Ting Dong, Shu-Bo Wan

**Affiliations:** 1 High-Tech Research Center of Shandong Academy of Agricultural Sciences, Ji’nan, China; 2 Shandong Provincial Key Laboratory of Crop Genetic Improvement, Ecology and Physiology, Ji’nan, China; 3 College of Life Sciences, State Key Laboratory of Crop Biology, Shandong Agricultural University, Tai’an, China; School of Medicine and Health Sciences, University of North Dakota, United States of America

## Abstract

Peanut is one of the calciphilous plants. Calcium (Ca) serves as a ubiquitous central hub in a large number of signaling pathways. The effect of exogenous calcium nitrate [Ca(NO_3_)_2_] (6 mM) on the dissipation of excess excitation energy in the photosystem II (PSII) antenna, especially on the level of D1 protein and the xanthophyll cycle in peanut plants under heat (40°C) and high irradiance (HI) (1 200 µmol m^−2^ s^−1^) stress were investigated. Compared with the control plants [cultivated in 0 mM Ca(NO_3_)_2_ medium], the maximal photochemical efficiency of PSII (Fv/Fm) in Ca^2+^-treated plants showed a slighter decrease after 5 h of stress, accompanied by higher non-photochemical quenching (NPQ), higher expression of antioxidative genes and less reactive oxygen species (ROS) accumulation. Meanwhile, higher content of D1 protein and higher ratio of (A+Z)/(V+A+Z) were also detected in Ca^2+^-treated plants under such stress. These results showed that Ca^2+^ could help protect the peanut photosynthetic system from severe photoinhibition under heat and HI stress by accelerating the repair of D1 protein and improving the de-epoxidation ratio of the xanthophyll cycle. Furthermore, EGTA (a chelant of Ca ion), LaCl_3_ (a blocker of Ca^2+^ channel in cytoplasmic membrane), and CPZ [a calmodulin (CaM) antagonist] were used to analyze the effects of Ca^2+^/CaM on the variation of (A+Z)/(V+A+Z) (%) and the expression of violaxanthin de-epoxidase (VDE). The results indicated that CaM, an important component of the Ca^2+^ signal transduction pathway, mediated the expression of the *VDE* gene in the presence of Ca to improve the xanthophyll cycle.

## Introduction

Plants are frequently subject to various environmental stresses. During summer, high temperature and high irradiance (HI) are the common stresses which plants are always faced with. Severe photo-oxidative damage to the photosynthetic apparatus is often attributed to the simultaneous occurrence of heat and HI and a decrease in photosynthesis often aggravates the amount of excess excitation energy [1]. Excess excitation energy, when not dissipated harmlessly, would be transformed to O_2_ to form reactive oxygen species (ROS) which could damage the photosynthetic apparatus, e.g. D1 protein, encoded by the *psbA* gene, can be used to reflect the degree of photoinhibition of PSII [2]–[4]. The repair of damaged PSII centers involves the degradation and *de novo* synthesis of this polypeptide in mature chloroplasts [5], [6]. This efficient repair mechanism is essential to maintain PSII in a functional state. Although the effects of exogenous calcium (Ca) on photosynthesis have been widely reported, its role on D1 protein under heat and HI stress requires further study.

During the long-term evolution, higher plants have developed many protective mechanisms to balance absorbed light energy with photosynthesis, thereby protecting the photosynthetic apparatus against photoinhibition [7]–[9]. The most important one is the xanthophyll cycle-dependent thermal energy dissipation, measured as the non-photochemical quenching (NPQ) of chlorophyll fluorescence [10]–[12]. This cycle comprises interconversions of three carotenoid pigments: violaxanthin (V), antheraxanthin (A), and zeaxanthin (Z), which are catalyzed by two enzymes: violaxanthin de-epoxidase (VDE: EC1.10.99.3) and zeaxanthin epoxidase (ZE: EC1.14.13.90). Under excess light conditions, VDE catalyzes the conversion of V to Z via A, whereas ZE catalyzes the reverse reaction [13]. Thermal dissipation of excitation energy is dependent on the accumulation of de-epoxidation products (A+Z) of the xanthophyll cycle [14], [15]. Furthermore, Z may directly protect the thylakoid membrane against photooxidation as an antioxidant [16], [17]. Thus, identifying mechanisms that can promote the xanthophyll cycle to alleviate the photoinhibition of PSII under excess light conditions is of great importance.

Ca^2+^ acts as a regulator of many physiological and biochemical processes in response to abiotic stresses in plants [18], [19]. Transient elevation of free Ca^2+^ in the cytoplast can be detected in plants in response to various stresses, such as high temperature [20], cold injury [21], drought stress [19], and salt stress [22]. The fact that Ca^2+^ improves plant resistance is related to maintaining a higher photosynthetic rate under stresses, and light-induced Ca^2+^ influx into chloroplasts not only influences the cytosolic concentration of free Ca^2+^ but also regulates the enzymatic processes inside the chloroplast [23]. Exogenous Ca^2+^ improves the net photosynthetic rate (Pn), carboxylation efficiency, and apparent quantum yield (AQY) of tobacco leaves under high temperature stress [24], and Ca^2+^ could also improve the Pn and Rubisco activity of cucumber at suboptimal temperatures [25]. The effect of Ca^2+^ on photosynthesis are attributed to the improvement of the stability of PSII reaction centers by enhancing the activity of antioxidant enzymes to alleviate ROS accumulation [24], [26]. For example, the superoxide anion, the initial product of photoreduction of O_2_, is dismuted by superoxide dismutase (EC 1.15.1.1, SOD) to H_2_O_2_ and O_2_ [27]. H_2_O_2_ is then converted into water by ascorbate peroxidase (EC 1.11.1.11, APX). Furthermore, exogenous Ca^2+^ could improve NPQ [28], which protects the photosynthetic machinery from inactivation and damage caused by excess irradiance [29]. NPQ has three components, among which fast component (qf) is the most important one, and is closely related to the xanthophyll cycle [30]. It has been reported that Z and possibly A, formed via the xanthophyll cycle, are involved in qf induction [10], [31]. However, the effects of exogenous Ca^2+^ on the xanthophyll cycle are yet to be reported.

Environmental signals trigger rapid and transient increases in cytosolic Ca^2+^. The alteration in the level of Ca^2+^ triggers a full range of signal transduction pathways through high-affinity, Ca-binding proteins such as calmodulin (CaM) [32], [33]. As a typical example of Ca relay protein, CaM binds Ca and regulates the activity of downstream target proteins [34]. However, whether this component is involved in the xanthophyll cycle-dependent energy dissipation under excess light energy remains unclear.

Peanut (*Arachis hypogaea* L.) remains one of the most important oil-crops in the world, and Ca is by far the most critical nutrient for this crop to achieve high yields. Ca deficiency in soil can results in a decrease in yield, e.g. unfilled pods. Photosynthetic products are the sources of peanut seeds, and peanut seedlings are usually subject to high temperature and HI in the field during peanut pod development. However, the effects of Ca^2+^ on peanut photosynthesis are yet to be reported. To explore the effect of exogenous Ca^2+^ on photoinhibition and the relationship between the expression of Ca-binding protein and the de-epoxidation state of the xanthophyll cycle, 0 mM Ca(NO_3_)_2_ and 6 mM Ca(NO_3_)_2_-treated peanut plants were used in our experiments combined with some Ca^2+^ inhibitors. It seemed that Ca^2+^ and Ca^2+^-dependent signal transduction pathways were apparently involved in the xanthophyll cycle to dissipate excess energy when peanuts were exposed to high temperature and HI stress.

## Materials and Methods

### Plant Material, Growth Conditions and Treatments

Peanut (*Arachis hypogaea* L.) cultivar “Huayu 22” was used in this study. The peanut plants were incubated with two revised Hoagland solutions, respectively, in which an equal volume of 0 or 6 mM Ca(NO_3_)_2_ (based on our previous study, 6 mM Ca(NO_3_)_2_ proved the best treatment in improving the heat and high irradiance stress of peanut plants) were presented. The seedlings incubated with 0 mM Ca(NO_3_)_2_ were marked as CK, and those incubated with 6 mM Ca(NO_3_)_2_ were marked as CA. The plants were grown at 25/20°C (day/night) under a 14 h photoperiod [300 µmol m^−2^ s^−1^ photon flux density (PFD)] for 20 d in a greenhouse. Functional leaves from plants were used in the experiments. To induce heat and high irradiance (HI) stress, with the adaxial side facing up, the detached leaves floating on the water were illuminated with 1 200 µmol m^−2^ s^−1^ PFD at high temperature (40°C).

### Determination of Ca^2+^ Content

Fresh leaf samples were dried in an oven at 105°C for 15 min, and then kept at 80°C to a constant weight. Approximately 150 mg of dried leaves was burned to ashes in an oven at 550°C. The ashes were dissolved in 65–68% HNO_3_ solution and diluted with 0.1 M HNO_3_ to 20 mL. Ca^2+^ was measured by an atomic absorption spectrometry (Hitachi Z-8000, Hitachi Ltd., Tokyo, Japan).

### Chlorophyll Fluorescence Measurement

Chlorophyll fluorescence was measured with a portable fluorometer (FMS2, Hansatech, UK) according to the protocol described by [35]. The initial fluorescence (Fo) was determined by modulated light (about 10 µmol m^−2^ s^−1^) which was low enough without inducing any significant variable fluorescence (Fv). The maximal fluorescence (Fm) was determined by 0.8 s saturating light of 8 000 µmol m^−2^ s^−1^ on a dark-adapted (adapted 15 min in darkness) leaf. The maximal photochemical efficiency (Fv/Fm) of PSII was calculated as Fv/Fm = (Fm-Fo)/Fm. NPQ was estimated as NPQ = Fm/Fm’-1 according to [36], where Fm was measured after dark adaptation for more than 2 h at room temperature prior to stress, Fm′ is the maximum intensity of fluorescence in light-acclimated leaves. NPQ can usually be divided into three different components according to their relaxation kinetics in darkness following a period of illumination [16]. The level of the fast relaxing quenching components of NPQ (qf) was highly correlated with the amount of Z and A synthesized via the xanthophyll cycle [16], and it was measured as described by [37].

### Thylakoid Membrane Preparation

Thylakoid membranes were prepared according to [38]. The leaves were homogenized in an ice-cold isolation buffer containing 400 mM sucrose, 50 mM HEPES-KOH, pH 7.8, 10 mM NaCl, 2 mM EDTA, and 2 mM MgCl_2_ and filtered through three layers of cheesecloth. The filtrate was centrifuged at 5 000×*g* for 10 min. The thylakoid pellets were washed with isolation buffer, re-centrifuged, and finally suspended in isolation buffer. The chlorophyll contents were determined spectrophotometrically as described by [39]. The resulting thylakoid membrane preparations were either used immediately or frozen in liquid N_2_ and stored at −70°C for use.

### SDS-PAGE and Western Blot Analysis

Thylakoid membrane proteins were denatured and separated using 12.5% polyacrylamide gradient gel that contained 6 M urea. 30 µg protein was applied to each well. The resolved protein was electroblotted to PVDF membrane and then probed with polyclonal antibodies raised in rabbits against the full-length D1 protein. The secondary antibody was peroxidase-conjugated goat anti-rabbit IgG. The D1 protein antibody was used at a dilution of 1∶500 and the secondary antibody was used at 1∶3 000.

### Determination of Reactive Oxygen Species

Hydrogen peroxide concentration was measured according to the method of [40] with modifications. The leaf samples (0.5 g) were homogenized with 3 mL phosphate buffer (50 mM, pH 6.8). The homogenate was centrifuged at 6 000×*g* for 25 min. Extracted solution (3 mL) was mixed with 1 mL of 0.1% titanium sulfate in 20% (v/v) H_2_SO_4_ and the mixture was then centrifuged at 6 000×*g* for 15 min. The intensity of the yellow supernatant was measured at 410 nm. H_2_O_2_ level was calculated using an extinction coefficient of 0.28 µmol^−1 ^cm^−1^ according to the standard curve plotted with known H_2_O_2_ concentration.

The assay for O_2_^•–^ was performed as described by [41]. Fresh leaves without midrib were thoroughly ground in an ice bath in a grinding medium containing 0.05 M phosphate buffer (pH 7.8). The homogenate was centrifuged at 5 000×*g* for 10 min at 4°C. The supernatant with phosphate buffer (pH 7.8) and 10 mM hydroxylammonium chloride was incubated at 25°C for 20 min, then 17 mM p-aminobenzene sulfonic acid and 7 mM α-naphthylamine were added, and the mixture was incubated at 25°C for 20 min. Finally, ethyl ether was added into the mixture that was centrifuged at 1 500×*g* for 5 min. The water phase was used to determine the absorbance at 530 nm. The O_2_^•–^ generation was calculated per g fresh mass of leaves.

### Pigment Analysis

Leaf disks were immersed in liquid N_2_ immediately after chlorophyll fluorescence measurement and stored at −70°C for use. Photosynthetic pigments were extracted from leaf disks with 80% ice-cold acetone. The extracts were centrifuged at 12 000×*g* for 5 min and supernatants were filtered through a 0.45 mm membrane filter before injection into reversed-phase high performance liquid chromatography (HPLC) using a Shimadzu Series model SCL-10AVP (Japan) equipped with an Elite Hypersil ODS2 4.6–250 mm cartridge column. Photosynthetic pigments were separated and quantified essentially following the method of [42]. The relative de-epoxidation state of the xanthophyll cycle pigments was calculated as (A+Z)/(V+A+Z) (%).

### Total RNA Extraction and Real-time Quantitive PCR (qRT-PCR) Analysis

Total RNA was extracted from the peanut leaves with the RNA simple kits (TIANGEN BIOTECH, China) according to the manufacture’s protocol. The DNase-treated RNA was reverse-transcribed using M-MLV reverse transcriptase (TIANGEN). Real-time RT-PCR was performed on the Bio-Rad CFX96TM Real-time PCR System using SYBR Real Master Mix (TIANGEN). The PCR thermal cycle conditions were as following: denature at 95°C for 1 min and 40 cycles for 95°C 10 sec; 61°C 30 sec; 68°C 20 sec. Peanut TUA5 was used as internal reference gene for calculating relative transcriptional levels.

Gene sequence of *AtpsbA, AhAPX, AhSOD,* and *AhCaM* were acquired from GenBank database, the accession number were X79898, EF165068, DQ499511, and AY517930, respectively. The amino acid sequence of *psbA* gene showed high identities among different species, thus the two degenerate primers were designed according to the sequence from *Arabidopsis thaliana*. Primer sequences were as follows: TUA5-F, 5′-CTGATGTCGCTGTGCTCTTGG-3′; TUA5-R, 5′-CTGTTGAGGTTGGTGTAGGTAGG-3′; psbA-F, 5′-ATCTGCTAATGAAGGTTACAG-3′; psbA-R, 5′-ATACCTACTACCGGCCAAG-3′; APX-F, 5′-TGCTGGAACTTTTGATGTGG-3′; APX-R, 5′-AACTACACCGGCCAACTG-3′; SOD-F, 5′-CAGTTCTTAGCAGCAGTGAG-3′; SOD-R, 5′-GGAACCCATGAAGACCAG-3′; CaM-F, 5′-GGTGCTCGACAAGGATCAA-3′; CaM-R, 5′-ACTCCTCGTAGTTGATCTGC-3′; VDE-F, 5′-TGCCTATGAAATCAGATGTGG-3′; VDE-R, 5′-CAAGTTTGTTGTCTTCTGTGTG-3′.

### Quantitative Analysis of CaM Protein

CaM concentrations in peanut leaves were determined with the Plant calmodulin (CaM) ELISA Kit (IBL, Germany) according to the manufacture’s protocol.

### Chemical Feeding

Some chemical agents were used to study the relationship between Ca^2+^ and the de-epoxidation ratio of xanthophyll cycle pigments. EGTA was used as a chelant of calcium ion, LaCl_3_ was used as a blocker of Ca^2+^ channel in cytoplasmic membrane and CPZ was used as a CaM antagonist. CA seedlings were pretreated with a daily spray of 5 mM EGTA, 2 mM LaCl_3_, and 0.05 mM CPZ for 6 days, respectively.

### Statistical Analysis

Statistical analyses were performed by analysis of variance (ANOVA) using SPSS version 13.0 (SPSS, Chicago, USA) and comparisons between the mean values were made by the least significant difference (LSD) at a 0.05 probability level.

## Results

### Ca^2+^ Content and Growth Analysis

**Figure 1:**
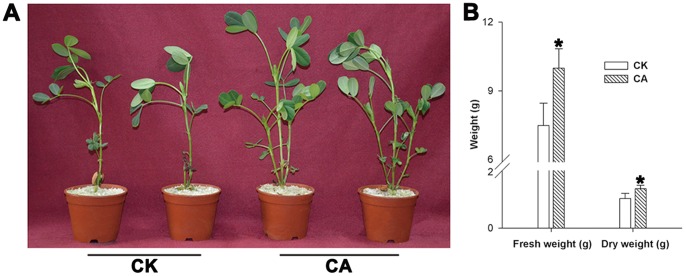
Effects of different Ca^2+^ concentrations on peanut seedlings’ growth. The seedlings were grown at 25/20°C (day/night) under a 14 h photoperiod [300 µmol m^−2^ s^−1^ photon flux density (PFD)] for 20 d in a greenhouse. Growth analysis (A), fresh weight and dry weight (B) of peanut plants treated with different Ca^2+^ concentrations. *Significant difference compared with CK using Student’s t-test at P<0.05.

Different Ca^2+^ culture media affected peanut seedling growth (Figure 1A). CK seedlings showed growth retardation relative to CA seedlings. This phenomenon was verified by the data of fresh weight and dry weight of all plants (Figure 1B). The fresh weight and dry weight of CK plants decreased obviously compared with 6 mM Ca(NO_3_)_2_-treated peanut plants. Meanwhile, the contents of Ca^2+^ in CA peanut leaves and roots were respectively higher by 31.7% and 40.9% compared with that in CK (Table 1).

**Table 1:** Effects of Ca(NO_3_)_2_ pretrearment on the Ca^2+^ content of peanut leaves and roots during heat and HI stress.

Ca(NO_3_)_2_ concentration (mM)	Ca^2+^ content (%)	
	Leaf	Root
0	1.01±0.01	0.22±0.03
6	1.33±0.02	0.31±0.02

The values are means ± SE of three independent experiments.

### Effects of Exogenous Application of Ca^2+^ on Photoinhibition of PSII under Heat and HI Stress

**Figure 2:**
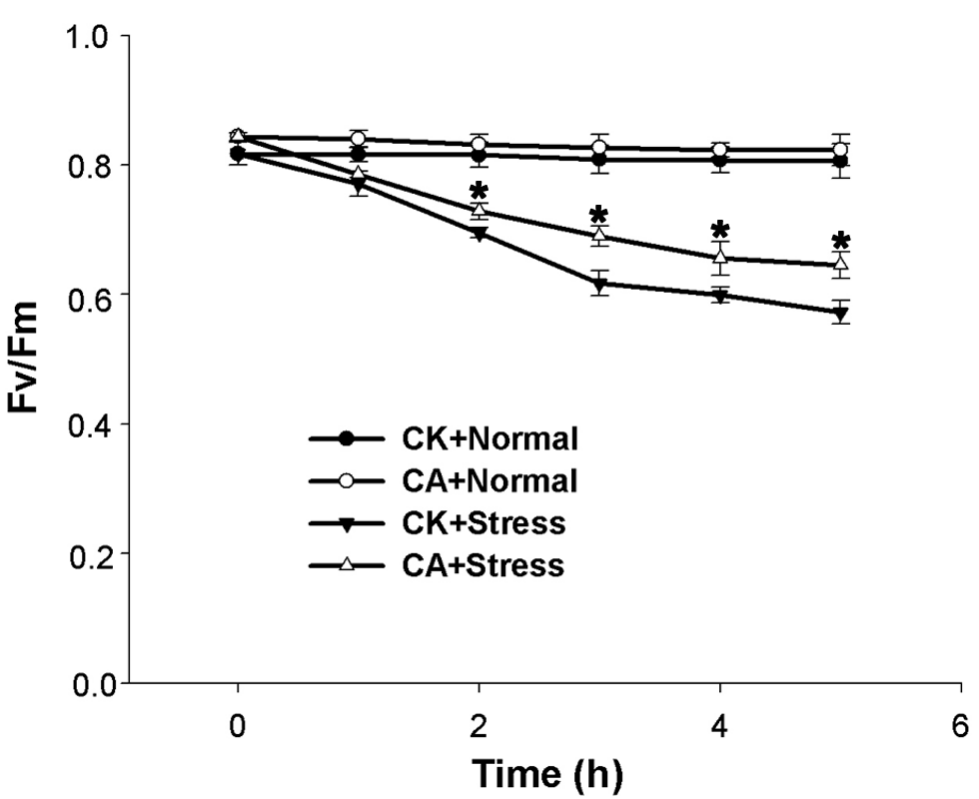
Effects of Ca^2+^ on the maximum photochemical efficiency of PSII (Fv/Fm) of peanuts. CK+Normal: 0 mM Ca(NO_3_)_2_-treated plants under normal growth conditions; CA+Normal: 6 mM Ca(NO_3_)_2_-treated plants under normal growth conditions, as a positive control; CK+Stress: 0 mM Ca(NO_3_)_2_-treated plants under heat (40°C) and high irradiance (1 200 µmol m^−2^ s^−1^ PFD) stress; CA+Stress: 6 mM Ca(NO_3_)_2_-treated plants under heat (40°C) and high irradiance (1 200 µmol m^−2^ s^−1^ PFD) stress. The data presented are the mean values ± SD of three individual experiments. *P* values were calculated by using t-test and are indicated by asterisks (*) when significantly different from CK+Stress (P<0.05).

The maximal photochemical efficiency of PSII (Fv/Fm) has been widely used as an indicator of photoinhibition. As shown in Figure 2, the Fv/Fm was not significantly changed in both CK and CA plants under normal conditions for 5 h. Upon exposure to heat and HI stress, Fv/Fm decreased in both CK and CA seedling leaves. The decrease in Fv/Fm was more evident in CK than in CA seedlings after 2 h of stress. At the end of the stress, Fv/Fm in CK and CA seedlings decreased by approximately 30.1% and 23.5% of their initial values, respectively. The results showed that under normal growth conditions, neither CK nor CA showed obvious photoinhibition, however, heat and HI stress induced more severe PSII photoinhibition in CK than in CA seedlings.

### Responses of D1 Protein Degradation to Heat and HI Stress in CK and CA Plants

**Figure 3:**
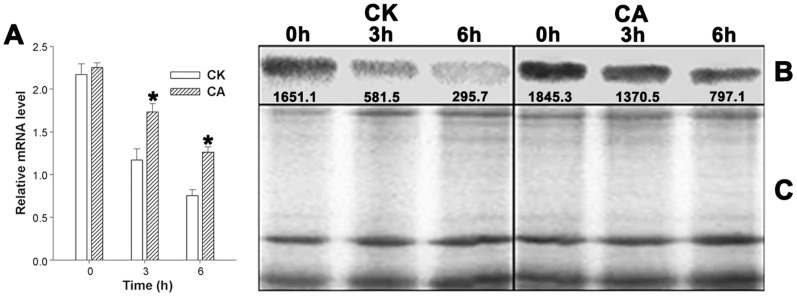
Analysis of D1 protein in peanut seedlings. qRT-PCR for psbA expression in peanut leaves of CK and CA before and after heat (40°C) and high irradiance (1 200 µmol m^−2^ s^−1^ PFD) treatment for 3 h and 6 h (A). *Significant difference compared with CK using Student’s t-test at P<0.05. Thylakoid membrane proteins were separated by SDS-PAGE and then probed with D1 antibody. Relative optical density (OD) has been added to ensure the difference in intensity (B). The same thylakoid membrane proteins were separated by SDS-PAGE and then stained with Coomassie brilliant blue R250 (C).

D1 protein, one of the major subunits of the PSII reaction center complex with rapid turnover, could be used to reflect the degree of photoinhibition. qRT-PCR and western blot analysis were performed to investigate the effects of the heat and HI stress on D1 protein at the levels of transcription and translation. Cross stress of heat and HI induced a decrease in both psbA expression (Figure 3A) and D1 protein content (Figure 3B) in all seedlings tested, but the decrease is less in CA than in CK plants.

### Effects of Exogenous Ca^2+^ on H_2_O_2_ and O_2_^•–^ Levels under Heat and HI Stress

**Figure 4:**
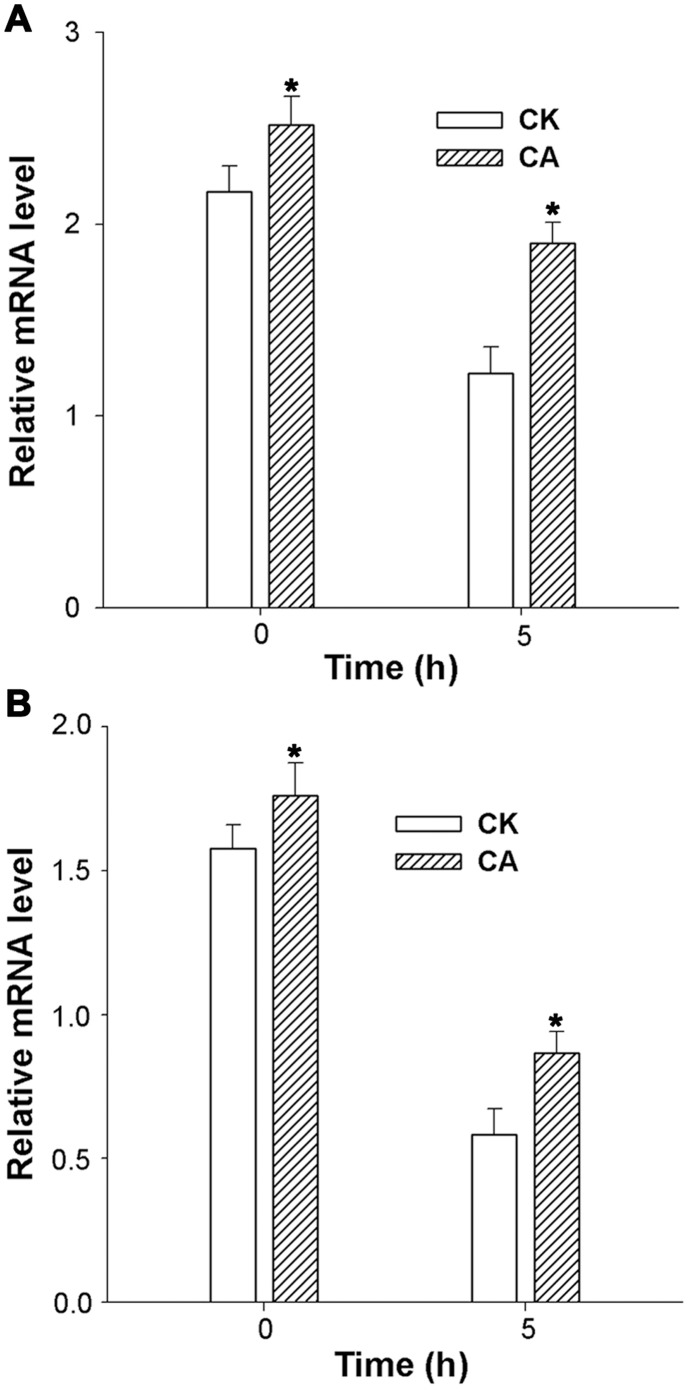
Expression of ROS-responsive genes in CK and CA plants. qRT-PCR for ascorbate peroxidase (APX) (A) and superoxide dismutase (SOD) (B) expression in CK and CA plants before and after heat (40°C) and high irradiance (1 200 µmol m^−2^ s^−1^ PFD) treatment for 5 h. The data presented are the mean values ± SD of three individual experiments. *P* values were calculated by using t-test and are indicated by asterisks (*) when significantly different from CK treatments (P<0.05).

To investigate whether enhanced PSII function is associated with less ROS accumulation under heat and HI stress, we examined the accumulation of H_2_O_2_ and O_2_^•–^. As shown in Table 2, the contents of H_2_O_2_ and O_2_^•–^ in the chloroplasts of all tested plants increased after the heat and HI stress. CK seedlings accumulated more H_2_O_2_ and O_2_^•–^ than those of CA seedlings after heat and HI stress for 5 h. Accordingly, higher expression of antioxidant enzymes APX (Figure 4A) and SOD (Figure 4B) in CA seedlings were observed in both normal conditions and heat and HI stress.

**Table 2:** H_2_O_2_ and O_2_^•–^ contents in the peanut leaves of CK and CA during heat and HI stress.

Ca(NO_3_)_2_ concentration (mM)	Treatment time	H_2_O_2_ (µmol g^−1^ FW)	O_2_^•–^ (nmol g^−1^ FW)
0	0 h	0.3489±0.0035	4.7105±0.014
	5 h	0.8128±0.0053	6.4710±0.049
6	0 h	0.3290±0.011	4.2410±0.036
	5 h	0.7189±0.0049	5.5712±0.15

The values are means ± SE of three independent experiments.

### Effects of Exogenous Ca^2+^ Application on Energy Dissipation in PSII under the Heat and HI Stress

**Figure 5:**
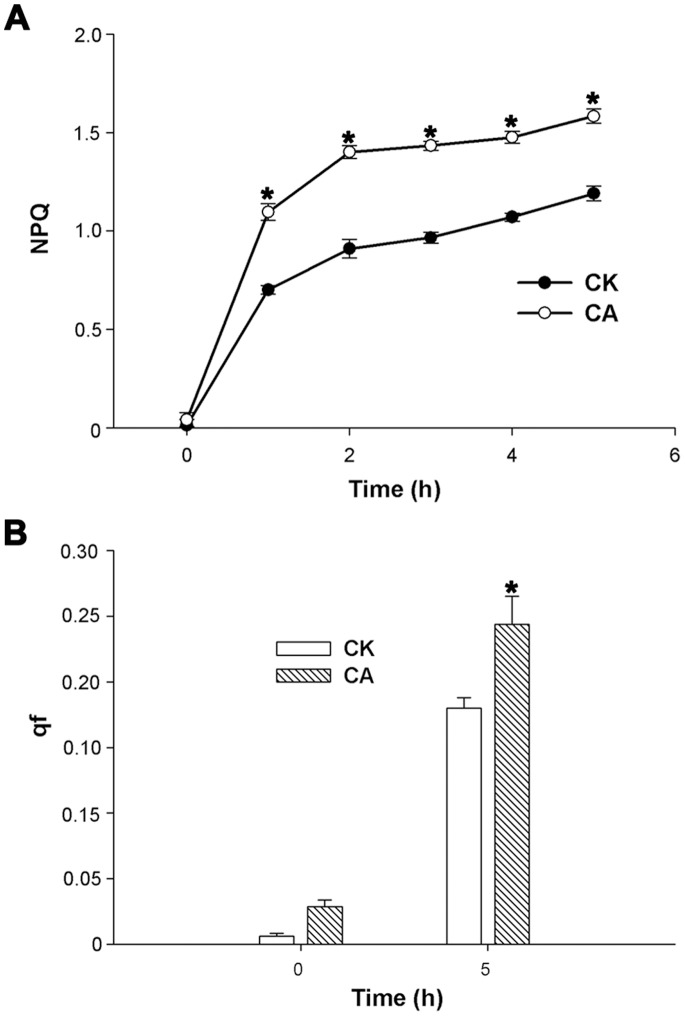
Characterization of NPQ and qf of CK and CA plants were monitored under normal and heat and HI stress. Effects of the heat (40°C) and high irradiance (1 200 µmol m^−2^ s^−1^ PFD) stress on non-photochemical quenching (NPQ) (A) and its fast relaxing component (qf) (B) in peanut seedling leaves. The data presented are the mean values ± SD of three individual experiments. *P* values were calculated by using t-test and are indicated by asterisks (*) when significantly different from CK treatments (P<0.05).

The capability for excess energy dissipation in PSII was investigated by determining NPQ. In both CK and CA seedling leaves, NPQ significantly increased when plants were exposed to heat and HI stress for 5 h. However, NPQ increased more rapidly in CA than in CK seedlings (Figure 5A).

After 5 h of stress, the qf of both CK and CA seedlings increased significantly, with the latter recording a higher increase (Figure 5B). This finding is consistent with the changes in NPQ during such stress (Figure 5A).

### Effects of Exogenous Ca^2+^ on the Xanthophyll Cycle under the Heat and HI Stress

**Figure 6:**
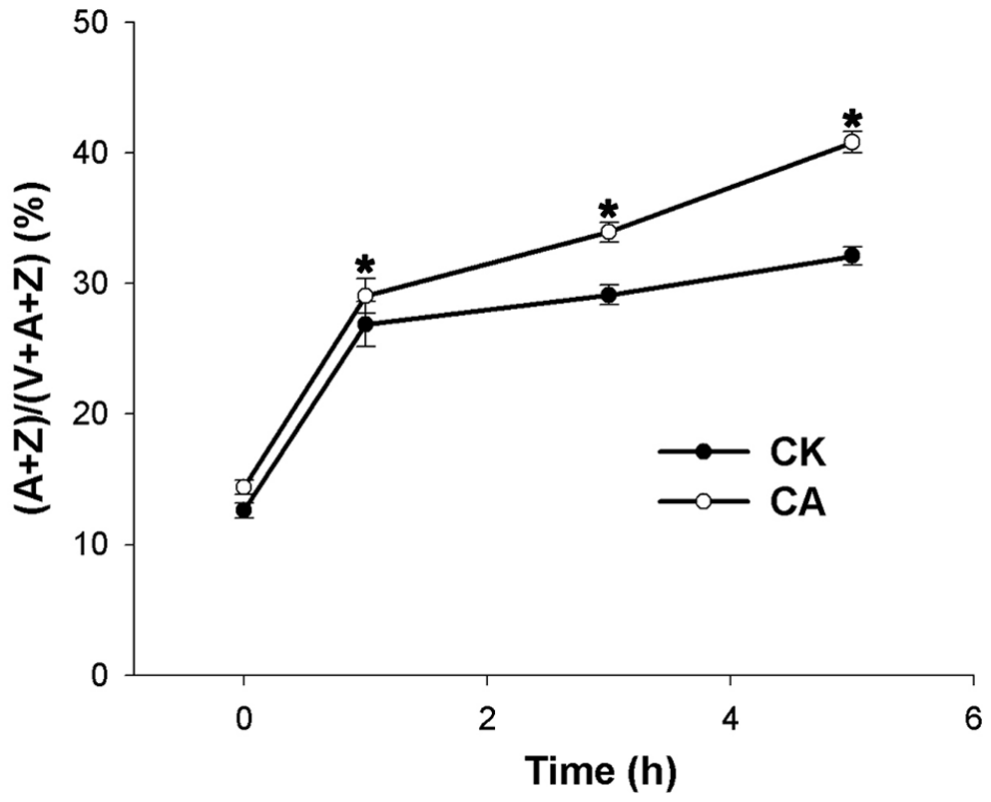
Function of calcium on the de-epoxidation ratio of the xanthophyll cycle after heat and HI treatments. Effects of the heat (40°C) and high irradiance (1 200 µmol m^−2^ s^−1^ PFD) stress on the de-epoxidation ratio of the xanthophyll cycle pigments (A+Z)/(V+A+Z) (%) in the CK and CA peanut seedlings. The data presented are the mean values ± SD of three individual experiments. *Significant difference compared with CK using Student’s t-test at P<0.05.

The total V+A+Z pool was not significantly changed in both CK and CA plants (data not shown), but the de-epoxidized ratio of the xanthophyll cycle pigments, (A+Z)/(V+A+Z), was higher in CA than in CK seedlings. At the end of the stress, the de-epoxidation level reached 32.1% and 40.8% in CK and CA seedlings, respectively (Figure 6). The accumulation of the xanthophyll cycle pigments A+Z was consistent with the values of NPQ and qf in both CK and CA leaves at the end of the stress (Figure 5).

### Analysis of CaM under Heat and HI Stress

**Figure 7:**
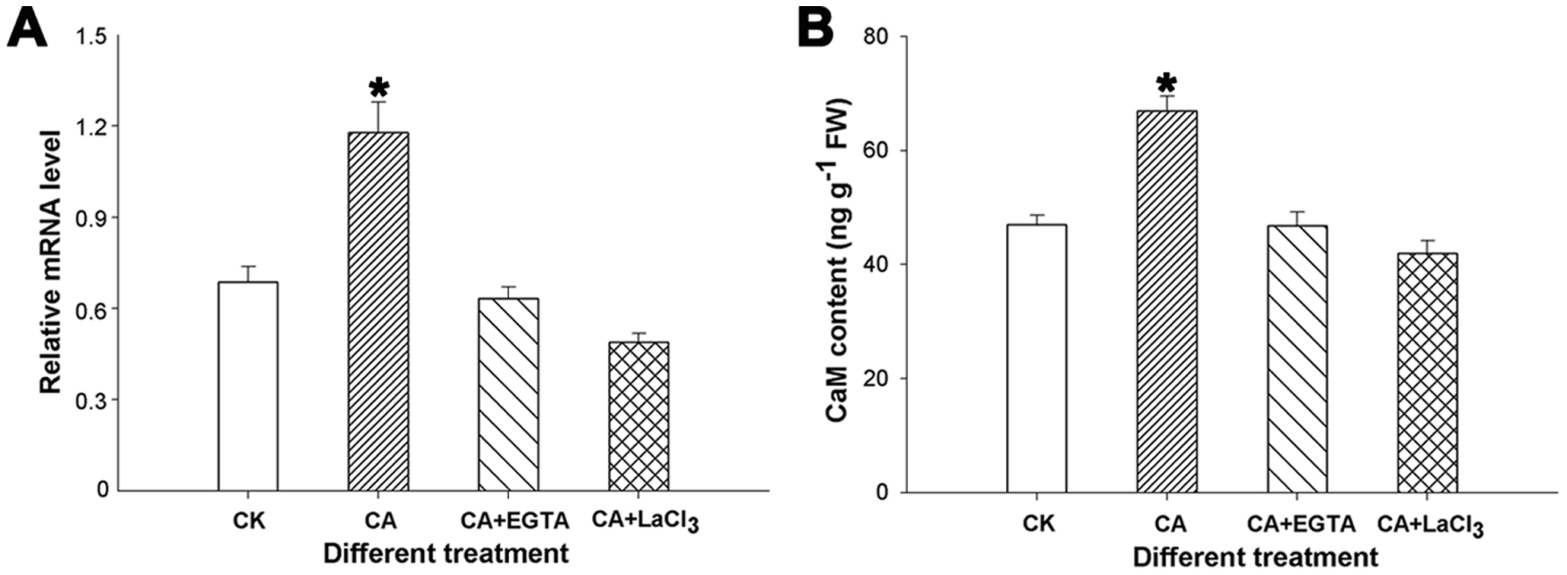
Analysis of calcium-binding protein calmodulin in leaf tissues. Expression of peanut *CaM* gene upon exposure to the heat (40°C) and high irradiance (1 200 µmol m^−2^ s^−1^ PFD) stress in different treated seedlings (EGTA and LaCl_3_) by qRT-PCR (A). Quantitative analysis of CaM protein upon exposure to the heat (40°C) and high irradiance (1 200 µmol m^−2^ s^−1^ PFD) stress in different treated seedlings (EGTA and LaCl_3_) (B). The data presented are the mean values ± SD of three individual experiments. *Significant difference compared with CK and chemical agents-treated plants using Student’s t-test at P<0.05.

In this study, the expression of the *CaM* gene and the protein level of CaM were analyzed in peanut leaves. After applying heat and HI stress for 5 h, either the transcriptional level (Figure 7A) or the translational level (Figure 7B) of CaM was obviously higher in CA than in CK seedlings. Meanwhile, the level of CaM was suppressed when CA seedlings were treated with EGTA and LaCl_3_ (Figure 7).

### The Relationship between Ca^2+^/CaM and the Xanthophyll Cycle under the Heat and HI Stress

**Figure 8:**
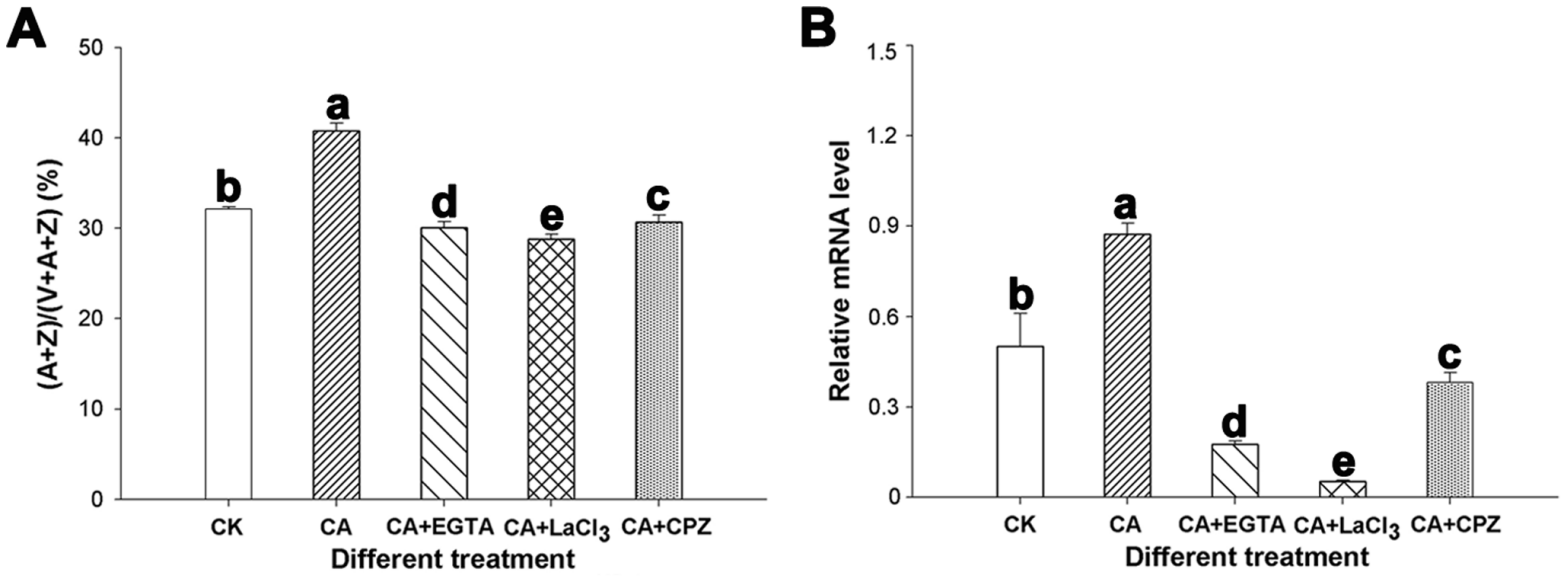
The relationship between Ca^2+^/CaM and the xanthophyll cycle under the heat and HI stress. The effect of EGTA, LaCl_3_, and CPZ on the de-epoxidation ratio of xanthophyll cycle (A) and the expression of *VDE* gene (B) after 5 h of heat (40°C) and high irradiance (1 200 µmol m^−2^ s^−1^ PFD) stress. The data presented are the mean values ± SD of three individual experiments.

EGTA, LaCl_3,_ and CPZ were used to assess the effects of different Ca-related components on the xanthophyll cycle. These three agents caused the decrease of the (A+Z)/(V+A+Z) ratio in CA leaves (Figure 8A), implying that the de-epoxidation state of the xanthophyll cycle was impaired in the Ca^2+^-deficient plants and that the Ca^2+^/CaM signal pathway had a stimulatory effect on the xanthophyll cycle.

To document the effects of Ca^2+^/CaM on the xanthophyll cycle further, the expression of the *VDE* gene was analyzed by qRT-PCR. We isolated and characterized the partial fragment of VDE using the cDNA prepared from peanut leaves according to the homologous sequences from other plants, and the sequence data have been deposited at the GenBank database under accession number JX914502. After 5 h heat and HI stress, as shown in Figure 8B, the transcriptional level of the *VDE* gene was drastically induced in the presence of Ca, an opposite result was caused by EGTA, LaCl_3,_ and CPZ.

## Discussion

In the natural environments, heat stress often occurs in combination with light stress. When light energy captured by antenna pigment could not be used efficiently, the excess energy would result in the photoinhibition of PSII. Although Ca^2+^ has been reported to play a role on alleviating oxidative stress and consequently improves photosynthesis under heat stress [24], the mechanism by which exogenous Ca^2+^ affects the dissipation of excess light, particularly the level of D1 protein and the xanthophyll cycle-dependent thermal energy dissipation under heat and HI stress, remains unclear. Studies on the photoprotection mechanisms of Ca^2+^ application will be useful in improving the tolerance of peanut to environmental stresses and ensuring high and stable yield of peanuts.

Ca is an essential macroelement for plant growth. A previous study has been suggested that Ca^2+^ deprivation causes the chlorosis and wilting of young spinach leaves, a decrease in photosynthesis, and a significant reduction of spinach plant weight [43]. Under Ca^2+^-deficit condition (Table 1), a similar phenomenon was also detected in the changes of the peanuts growth performance (Figure 1). Upon exposure to heat and HI stress, exogenous Ca^2+^ alleviated PSII photoinhibition of peanut seedlings (Figure 2) and kept higher D1 protein expression at the levels of transcription and translation (Figure 3), which implied that Ca^2+^ was involved in some mechanisms protecting photosynthetic apparatus of peanut seedlings from such stress. More severe PSII photoinhibition (Figure 2) and lower level of D1 protein (Figure 3B) in CK seedlings might be related to the damage caused by the accumulation of excess energy in photosynthetic apparatus [44]. Under excess energy conditions, it will bring about oxidative damage when the balance between ROS production and ROS scavenging was disrupted [11].

Exposure of leaves to heat and HI stress induces an imbalance between energy supply and utilization in chloroplasts, resulting in an increased excitation pressure on PSII and the production of more ROS, especially under Ca^2+^-deficit condition (Table 2). ROS could attack the sensitive site of PSII and suppress the repair cycle of the photodamaged D1 protein by inhibiting the process of peptide elongation [45], [46]. However, plants have developed a series of both enzymatic and non-enzymatic detoxification systems to counteract ROS, thereby protecting cells from oxidative damage. Ca^2+^ is required for the maintenance of antioxidant activity under heat stress [47]. It can activate some antioxidant enzymes directly [48], [49] or indirectly by binding CaM to plant catalases [50]. In this study, it is obvious that exogenous Ca^2+^ could alleviate the accumulation of ROS (Table 2) by improving the antioxidant enzyme expression (Figure 4).

When exposed to photoinhibition, the xanthophyll cycle-dependent NPQ for higher plants is the most useful mechanism to dissipate excess energy. NPQ is induced in the presence of *Δ*pH and functions in the dissipation of excess photon energy against photosynthesis as heat [51], alleviating the damage caused by excitation energy to PSII. Previous research mainly focused on the effects of high light stress or chilling stress with low light on the xanthophyll cycle in the model plants [52], [53] through its participation in NPQ. Under N limitation, the xanthophyll cycle-dependent thermal dissipation was also enhanced in leaves of maize [54], spinach [55], and apple [56]. However, the function of Ca^2+^ in crop plants in response to heat and HI stress-induced photoinhibition is still unclear. In our study, both NPQ and the ratio of (A+Z)/(V+A+Z) increased in CK and CA seedlings when exposed to the heat and HI stress, thus the energy dissipation and the xanthophyll cycle were both inhibited in Ca^2+^-deficit seedlings (Figures 5A, 6). Among three components of NPQ, qf is the main component of NPQ and is highly correlated with the amount of Z and A synthesized via the xanthophyll cycle [16]. Aside from the establishment of a pH gradient across the thylakoid membrane, the accumulation of Z or A in excess light depending on the activity of the enzyme VDE was also essential for the generation of qf [57]. The higher qf (Figure 5B) and higher de-epoxidation ratio of the xanthophyll cycle (Figure 6) in Ca^2+^ application seedlings suggested that Ca^2+^ could improve the xanthophyll cycle-dependent energy dissipation. To the best of our knowledge, the results of the present study are the first evidence of a relationship between Ca and the xanthophyll cycle in peanuts under the heat and HI stress.

Thus, it can be concluded that Ca^2+^ application for peanut seedlings could improve crop’s resistance to heat and HI stress by improving the xanthophyll cycle and ROS scavenging system. Moreover, the opposite effects of Ca^2+^-deficit cultivation may be related to lower membrane stability and integrity and may lower some enzymes’ activities because Ca^2+^ is believed to be involved in the formation of biological membrane and in the activation of some enzymes [58], [59]. However, a question remains on the effects of Ca^2+^-mediated signal transduction pathway on excess energy dissipation, especially the interconversions of the three pigment components of the xanthophyll cycle.

As a second messenger in plants, Ca^2+^ plays a pivotal role in signal transduction pathway under abiotic stress. Several families of Ca^2+^ sensors have been identified in higher plants. CaM and CaM-related proteins are the best known ones, which sense the “Ca signature” and participate in Ca^2+^-mediated signal transduction pathway and gene regulation during stress responses [60]. The heat and HI stress improved the expression of the *AhCaM* gene in peanut leaves under Ca^2+^ application, and its expression was inhibited after the addition of EGTA and LaCl_3_ (Figure 7A), which indicates that the *AhCaM* gene could be induced by the heat and HI stress in a Ca-dependent manner. Analysis of CaM at the protein level also verified that Ca^2+^ plays a role in resisting to heat and HI stress through Ca^2+^/CaM signal transduction pathway (Figure 7B). Additionally, the (A+Z)/(V+A+Z) ratio increased under Ca^2+^ application, but was inhibited in CK, especially in EGTA, LaCl_3,_ and CPZ-treated plants (Figure 8A), which was further confirmed by the expression of the *VDE* gene (Figure 8B). That is, exogenous Ca^2+^ can improve the de-epoxidation state of the xanthophyll cycle through the Ca^2+^/CaM-mediated stimulation of the *VDE* gene. CaM is a small acidic protein that is primarily expressed in the cytoplasm. However, CaM has been found to be present in several organelles in plants, such as the nucleus and chloroplasts [61], as well as in the extracellular matrix [62]. In addition, CaM-regulated proteins exist in the extracellular matrix, nucleus, and chloroplasts [62]–[64]. However, the mechanism by which CaM directly or indirectly affects the activity of chloroplastic enzyme VDE remains unclear and is an area for further study.
